# Gender differences in pleasure: the mediating roles of cognitive flexibility and emotional expressivity

**DOI:** 10.1186/s12888-022-03945-9

**Published:** 2022-05-05

**Authors:** Chunyu Wang, Zhihao Zhang, James Allen Wiley, Tingting Fu, Jin Yan

**Affiliations:** 1 Xiangya Nursing School, Central South University, Changsha, Hunan China; 2 Institute for Health Policy Studies, UCSF School of Medicine, San Francisco, CA USA; 3 School of Public Administration, Central South University, Changsha, Hunan China; 4 Department of Nursing, The Third Xiangya Hospital of Central South University, NO.138 Tong Zipo Road, Changsha, 410000 Hunan China

**Keywords:** Gender differences, Anticipatory pleasure, Consummatory pleasure, Cognition flexibility, Emotional expressivity

## Abstract

**Background:**

Gender differences have been found to be associated with individuals’ pleasure. Cognitive flexibility and emotional expressivity might play an important role between gender differences and pleasure. This current study is to explore the mediating role of cognitive flexibility and emotional expressivity in the relationship between gender differences and pleasure.

**Method:**

In this cross-sectional study, a sample of 1107 full-time university students from five colleges in Tianjin, Chinese mainland was investigated by questionnaire. All participants completed the Temporal Experience of Pleasure Scale (TEPs), the Cognitive Flexibility Inventory (CFI), and the Berkeley Expressivity Questionnaire (BEQ).

**Results:**

The results of independent T-test suggested that females reported better emotional expressivity, anticipatory pleasure and consummatory pleasure than males, whereas males had better cognitive flexibility than females. Using bootstrapping approach revealed that the partially mediation effects of cognitive flexibility on gender differences in anticipatory and consummatory pleasure, and that of emotional expressivity on gender differences in anticipatory and consummatory pleasure. Results of this present study stated that cognitive flexibility and emotional expressivity play a partial mediating role in explaining gender differences in anticipatory and consummatory pleasure.

**Conclusion:**

Females had higher anticipatory and consummatory pleasure because they tend to use emotional regulation strategy to express their emotion.

## Introduction

Anhedonia is defined as individuals markedly diminished interest or pleasure in all or almost all, activities most of the day, nearly every day [1], which is a core feature of depression. Approximately 74% of depressed adolescents reported anhedonia [2], and the greater anhedonia leads to the higher happen of suicide behavior among children [3] and adolescents [4]. Current drugs and mainstream psychotherapy for treating this disorder are not effective. From the perspective of positive psychology, carrying out relevant researches on pleasure has a strong theoretical and practical significance for preventing individual anhedonia and promoting healthy behaviors.

Pleasure is not only a sensory event, but also a subjective hedonic experience related to incentivizing or rewarding [5]. Klein [6] divided pleasure into two dimensions: anticipatory pleasure and consummatory pleasure. Anticipatory pleasure regards as predicting a targeting future reward and having a reward motivation and goal-directed behavior. On the contrary, consummatory pleasure is an “at the moment” pleasure in the process of reward attainment [7, 8]. Gard et al. [9] found that females had higher levels of anticipatory pleasure and consummatory pleasure than males. From our literature review, we found that sociologists have already explored gender differences in pleasure from societal conditions, gender equality, and religiosity [10]. Whereas the psychological mechanism that creates gender differences in pleasure is still not clear [11].

According to Mayer [12] and Salovery’s the Emotional Intelligence theory, regulating emotion, one of the important components of emotional intelligence, could up-regulate positive emotions and down-regulate negative emotions [13]. Cognitive reappraisal and expressive suppression are two common and effective individual emotional regulation strategies [14]. On the one hand, cognitive flexibility was the foundation of cognitive reappraisal ability [15]. It refers to the ability to transform cognitive sets effortlessly according to the changes of the environmental stimuli [16]. Individuals who have higher cognitive flexibility can quickly change their minds to cope with such challenges, keep a positive situation and higher life satisfaction [17]. A recent study indicated that, due to gender differences in brain regions, especially the hippocampus, females showed a greater prevalence of disease and more severe decreased cognitive symptoms in depression [18]. Females who suffered from depression tend to lost interest and pleasure [2]. We hypothesized that (h1) cognitive flexibility might play a mediating role between gender differences and anticipatory and consummatory pleasure.

Emotional experience refers primarily to external events or stimuli evoked by individual physiological arousal, and emotional expressivity is the external expression of subjective experience or thinking [8], the behavior of emotional expressivity that is about to happen or is happening, commonly is controlled by emotional suppression [19]. Regulating emotions through suppressing emotions in the long term may produce mood disorders [20]. Therefore, emotional expressivity is a more positive and healthy way to regulate emotions. Emotional expressivity has been conceptualized as the transformation of an individual behavior related to emotional experiences, such as smile, laugh, cry, frown and vent [21]. Individuals who are good at expressing their emotions have better relationships, higher well-being, and lower experience of anxiety, depression and guilty [22]. Studies presented females had higher emotional expressivity than males [23, 24]. We hypothesized that (h2) emotional expressivity might play a mediating role between gender differences and anticipatory and consummatory pleasure.

The aims of this study were to explore how cognitive flexibility and emotional expressivity influence gender differences in anticipatory and consummatory pleasure from a psychological perspective.

## Methods

### Procedure and participants

This is a cross-sectional study involving college students from five full-time undergraduate universities in Tian Jin, China. Convenience sampling was used to recruit 1107 Chinese college students. Based on the class group and with the assistance of the class instructors, the questionnaires are distributed in a centralized manner. During the survey, the questionnaires are collected by systematically trained researchers with a psychology background, and all the questionnaires are collected on the spot. For the participants, inclusion criteria were as follows: (1) taking part in the present study on their willingness; (2) full-time university student. Exclusion criteria were: (1) having or had self-reported mental and cognitive disorders or brain injury; (2) evidence of substance abuse or dependence in the past 3 months; and (3) having or had psychological counseling and treatment.

### Measures

#### Pleasure

The 20-item Temporal Experience of Pleasure Scale (TEPs) is a self-report instrument that assesses anticipatory and consummatory pleasure experience [9]. Pleasure was measured by asking participants “I look forward to many things in life”. We used the Chinese version of the Temporal Experience of Pleasure Scale, a self-report evaluating the happiness of college students [25]. The Chinese version of TEPs is a 19 item, 6-point Likert from 1 (very false) to 6 (very true). The Chinese version has four factors: abstract anticipatory pleasure, contextual anticipatory pleasure, abstract consummatory pleasure and contextual consummatory pleasure. A higher total score reflects higher levels of pleasure and lower levels of the anhedonia. In the current study, the Cronbach’s alpha for the TEPs is 0.85.

#### Cognitive flexibility

The 20-item cognitive flexibility inventory (CFI [26]) is used to measure the participants’ cognitive flexibility level. Cognitive flexibility was measured by asking participants “I am good at analyzing and evaluating a variety of situations and situations”. The Chinese version of Cognitive Flexibility Inventory (CFI) was used in this study. The Chinese version of CFI consists of 20 items, each with a range from 1 to 5 (from 1 = never to 5 = frequently). Higher scores reflect the higher degree of cognitive flexibility (Dennis et al., 2010). The Chinese version of CFI has been demonstrated to have good reliability and validity in Chinese populations [18]. In the current study, the Cronbach’s alpha for the CFI is 0.89.

#### Emotional expressivity

The 16-item Berkeley Expressivity Questionnaire (BEQ) was used to measure an individuals’ emotional expressivity [21]. Emotional expressivity was measured by asking participants “When I’m happy, I show it”. The Chinese version of BEQ was validated in Chinese college students with good reliability and validity [27]. The Chinese version of BEQ was used in this study. The Chinese version of BEQ consists of 16 items, and contains five categories including positive expressivity, negative expressivity, negative inhibition, positive impulse strength and negative impulse strength. Higher scores indicate greater emotional expressivity [24]. In the current study, the Cronbach’s alpha for the BEQ is 0.83.

### Statistical analysis

In this study, the IBM SPSS 24.0 computer software was used for statistical analysis. Independent T-test were used to determine whether there were any significant differences among anticipatory pleasure, consummatory pleasure, cognitive flexibility and emotional expressivity. Pearson’s correlations analysis was used to examine the associations of cognitive flexibility, emotional expressivity, anticipatory and consummatory pleasure. Multiple regression analysis was performed to test the mediating role of cognitive flexibility emotional expressivity. The mediation model was calculated by Mplus 8.1, applying with 95% bias corrected confidence interval (CI) based on 1000 bootstrap samples. It was used to examine the indirect effects of gender differences in anticipatory and consummatory pleasure through cognitive flexibility and emotional expressivity. The effects were considered as statistically significant if the 95% CI did not contain zero. Gender (0 = females, 1 = males) served as independent variable, cognitive flexibility and emotional expressivity as mediator, anticipatory pleasure and consummatory pleasure were dependent variables in this model. Values of *p* < 0.05 were considered significant.

### Common methods Bias test

The result of Harman’s Single-Factor test states that there were 12 factors with eigenvalues greater than one. The explanation amount of variation of the first factor was only 16.66%, which is lower than 40% threshold. It shows that the common methods bias test is not obvious.

## Results

### Preliminary analyses

Descriptive statistics were presented in Table 1. In all the samples, the ages of the 1107 participants ranged from 18 to 24 years (*M*_*age*_ = 20; *SD* = 1.73). Among them, 422 are male and 685 are female. And 39.40% of the participants were from urban areas, 60.60% were from rural areas. The results of independent T-test suggested that there were significant gender differences in anticipatory pleasure (*t* = − 6.03, *p* < 0.001) and consummatory pleasure (*t* = − 4.23, *p* < 0.001) --Females had both higher anticipatory and consummatory pleasure. Analyses also indicated that males exhibited slightly higher level of cognitive flexibility compared to females (*t* = 2.69, *p* < 0.01), and females exhibited slightly higher level of emotional expressivity compared to males (*t* = − 4.57, *p* < 0.001). Cognitive flexibility was positively correlated with anticipatory pleasure (*r* = 0.11, *p* < 0.001) and consummatory pleasure (*r* = 0.27, *p* < 0.001). The positive correlation was also found between emotional expressivity and anticipatory pleasure (*r* = 0.44, *p* < 0.001) and consummatory pleasure (*r* = 0.35, *p* < 0.001).

**Table 1:** Descriptive statistics and correlations among study variables

	1	2	3	4
1.Anticipatory pleasure	1			
2.Consummatory pleasure	0.64^***^	1		
3.Cognitive flexibility	0.11^***^	0.27^***^	1	
4.Emotional expressivity	0.44^***^	0.35^***^	−0.05	1
Male	35.48 ± 6.17	43.89 ± 7.65	72.64 ± 9.65	68.03 ± 11.87
Female (M ± SD)	37.82 ± 6.35	45.82 ± 7.16	71.09 ± 9.04	71.33 ± 11.53
T-tests	−6.03^***^	−4.23^***^	2.69^**^	−4.57^***^

^*^ *p* < 0.05 ^**^ *p* < 0.01 ^***^p < 0.001

### Simple mediation model

**Fig. 1:**
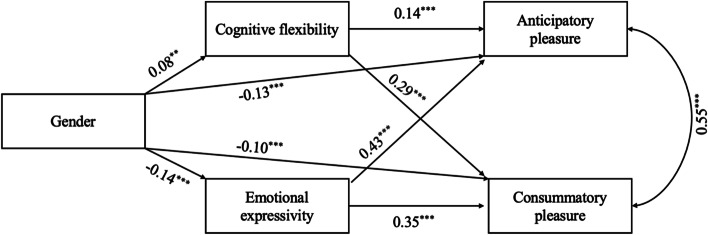
Regression coefficients of the parallel mediation model of cognitive flexibility and emotional expressivity in the association between gender differences and anticipatory and consummatory pleasure. (^*^ *p* < 0.05, ^**^ *p* < 0.01, ^***^p < 0.001)

As shown in Fig. 1, the path model implemented in Mplus 8.1 was used to examine the theoretical model. All the path coefficients (showed in Fig. 1) for the indicators on the variables were statistically significant (*β* = −.14 ~ 0.35, *p* < 0.01). The structural model had an excellent fit to the date: χ^2^ = 983.94(*n* = 10), CFI = 0.99, TLI = 0.99, RMSEA = 0.03, SRMR = 0.01. Linear regression (Table 2 and Table 3) was performed with cognitive flexibility/emotional expressivity as dependent variables and gender differences (females = 0, males = 1) as independent variables. Gender differences were positively associated with cognitive flexibility (*β* = .08, *p* < 0.01), accounting for a 0.6% of the variance (R^2^ = .006). Gender differences were negatively associated with emotional expressivity (*β* = −.14, *p* < 0.001), accounting for a 1.8% of the variance (R^2^ = .018). Linear regression was performed with pleasure as dependent variables and gender differences/cognitive flexibility/emotional expressivity as independent variables. The effect of gender differences (*β* = −.13, *p* < 0.001), cognitive flexibility (*β* = 0.14, *p* < 0.001) and emotional expressivity (*β* = 0.43, *p* < 0.001) in anticipatory pleasure were significant, accounting for a 22.4% of the variance (*R*^2^ = .224). The effect of gender differences (*β* = −.10, *p* < 0.001), cognitive flexibility (*β* = 0.29, *p* < 0.001), and emotional expressivity (*β* = 0.35, *p* < 0.001) in consummatory pleasure were significant, accounting for a 21% of the variance (*R*^2^ = .210).

**Table 2:** Regression analysis results of cognitive flexibility and emotional expressivity

	Cognitive flexibility			Emotional expressivity		
	β	SE	*t*	β	SE	*t*
Gender difference	0.081	0.574	2.687**	−0.136	0.722	−4.569***
△R2	0.006			0.018		
△F	7.219**			20.875***		

** *P* < 0.01, *** *P* < 0.001

**Table 3:** Regression analysis results of anticipatory pleasure and consummatory pleasure

	Anticipatory pleasure			Consummatory pleasure		
	β	SE	*t*	β	SE	*t*
Gender difference	−0.131	0.352	−4.905***	−0.103	0.412	−3.791***
Cognitive flexibility	0.140	0.018	5.276***	0.294	0.021	10.94***
Emotional expressivity	0.428	0.015	15.992***	0.374	0.017	12.86***
△R2	0.224			0.210		
△F	107.588***			98.899***		

** *P* < 0.01, *** *P* < 0.001

The direct and indirect effects of gender differences in pleasure are presented in Table 4. The bootstrapped estimation procedure (with 1000 bootstrapping samples) was adopted to test the mediating effect. Results showed that gender differences had indirect effects on anticipatory pleasure (indirect effect = 0.01, effect size = 5.56%, *CI*_*95%*_ = 0.09 to 0.20, *p* < 0.05) and consummatory pleasure (indirect effect = 0.02, effect size = 15.38%, *CI*_*95%*_ = 0.24 to 0.35, *p* < 0.01) through cognitive flexibility, which were both significant. Simultaneously, our analysis displayed that the indirect effect from gender differences to anticipatory pleasure (indirect effect = − 0.06, effect size = 33.33%, *CI*_*95%*_ = 0.37 to 0.48, *p* < 0.001), and consummatory pleasure (indirect effect = − 0.05, effect size = 38.46%, *CI*_*95%*_ = 0.29 to 0.40, *p* < 0.001) via emotional expressivity was significant. Moreover, the direct effect of gender differences in anticipatory pleasure and consummatory pleasure was significant. These results suggested that cognitive flexibility and emotional expressivity partly mediated the relationship of gender differences in both anticipatory and consummatory pleasure.

**Table 4:** Bootstrapping indirect effect and 95% confidence intervals (CI) for the mediating model

				95% CI	
Effect	Model pathways	Adjusted indirect effect	Effect size	Lower	Upper
Direct effect	Gender→Anticipatory pleasure	− 0.13	72.22%		
	Gender→Consummatory pleasure	−0.10	76.92%		
Indirect effect	Gender→Cognitive flexibility→Anticipatory pleasure	0.01	5.56%	.09	0.20
	Gender→Cognitive flexibility→Consummatory pleasure	0.02	15.38%	0.24	0.35
	Gender→Emotional expressivity→Anticipatory pleasure	−0.06	33.33%	0.37	0.48
	Gender→Emotional expressivity→Consummatory pleasure	−0.05	27.78%	0.29	0.40
Total indirect effect	Indirect effect of anticipatory pleasure	−0.05	23.08%		
	Indirect effect of consummatory pleasure	−0.03			
Total effect	Total effect of anticipatory pleasure	−0.18			
	Total effect of consummatory pleasure	−0.13			

## Discussion

### Summary

The purpose of this study was to explore whether gender differences in anticipatory and consummatory pleasure can be explained by cognitive flexibility (h1) and emotional expressivity (h2). Our results showed that females (37.82 to 45.82 ﻿ ± 6.35 to 7.16) had both higher anticipatory and consummatory pleasure than males (35.48 to 43.89 ﻿ ± 6.17 to 7.65). At the same time, the results of this study certified our hypotheses—through the mediating role of cognitive flexibility and emotional expressivity, gender differences have a prediction on individual anticipatory and consummatory pleasure. In this Chinese college students, males (72.64 ﻿ ± 9.65) had higher cognitive flexibility than females (71.09 ﻿ ± 9.04), while females (71.33 ﻿ ± 11.53) had higher levels of emotional expressivity than males (68.03 ﻿ ± 11.87).

### Cognitive flexibility and pleasure

In this study, males (72.64 ﻿ ± 9.65) have higher cognitive flexibility than females (71.09 ﻿ ± 9.04) in both anticipatory and consummatory pleasure, which is consistent with other studies. In Demirtaş [28]‘s study, individuals who had higher cognitive flexibility were good at utilizing kinds of coping strategies to deal with any problems and then reduce their negative emotions. In addition, males with higher cognitive flexibility are easier to realize how to control challenges of their life events, choose the fit solutions to settle, remove their own incorrect thoughts and further increase well-being [29]. People who have higher cognitive flexibility are more extraverted, opening and self-restrained, so that have more pleasure [30]. Therefore, compared to females, males mainly use their cognitive flexibility to understand events from multiple angles to produce and strengthen their own pleasure experience.

### Emotional expressivity and pleasure

In line with most of previous study [31], our results demonstrated that, females (71.33 ﻿ ± 11.53) have a higher emotional expressivity than males (68.03 ﻿ ± 11.87) in both anticipatory and consummatory pleasure. It indicates females are easy to express their emotions no matter it is positive or negative stimuli. A meta-analysis indicated that, compared to males, females were easier to cry [32] and the average smile numbers of females are also more than males [33]. A study showed that individuals who were good at expressing their positive emotions could acquire more social support and cooperation, decrease their social loneliness and depression [34], and further actually increase individual positive emotions at present. Scheff [35] stated that expressing emotion is the intuitive human response and a way to address tragic experience, such as crying. In addition, individuals who are good at expressing positive emotions have a strong ability of self-regulation [36]. That could effectively avoid conflict, promote the ability of problem-solving, strengthen cooperation with each other and promote self-willing behaviors [37]. Thus, compared to males, females mainly use their emotional expressivity to vent negative emotions, then produce and strengthen their pleasure.

### The mediating model of cognitive flexibility and emotional expressivity

This study found that (1) gender differences can positively predict pleasure, and (2) cognitive flexibility (indirect effect = 0.01to 0.02, effect size = 5.56 to 15.38%, *CI*_*95%*_ = 0.09 to 0.35, *p* < 0.05) and emotional expressivity (indirect effect = − 0.06 to − 0.05, effect size = 33.33 to 38.46%, *CI*_*95%*_ = 0.09 to 0.35, *p* < 0.001) play a partial mediating role between gender differences and anticipatory and consummatory pleasure. Males have higher cognitive flexibility and females have higher emotional expressivity. However, females had higher anticipatory and consummatory pleasure. Therefore, the mediating effect of emotional expressivity is larger. In other words, females feel more joyful than males, which may be due to females’ better use of emotion regulation strategy of emotional expressivity played a more vital role. When males face stressful life events or other negative stimuli, due to the higher cognitive flexibility of the male group, males can gradually understand and reappraise afterward—plays a certain “compensation” role and further relieves their own negative emotions and pain. However, females are better at using emotion expressivity strategies to adjust their own emotions. Once they face negative events, they will aggravate their own negative emotions—play an “amplification” role, which may lead to depression or other emotional disorders. This also explains, to a certain extent, why females experience depression approximately twice as many as males [38] and women are more susceptible to depression. It is able to modify the individual pleasure experience through increasing cognitive flexibility and positively expressing emotion to treat depression. For example, lots of studies showed cognitive-behavioral therapy (CBT) is an effective way for treating depressed people, which contains activating behavior and restructuring cognition [39–41]. Researchers should pay more attention to such training programs.

### Limitation

This study has some limitations. First, all the reports came from undergraduates, and future research should expand the study population, including people of different ages. Second, this study is a cross-sectional study and lacks longitudinal follow-up studies, so it is not possible to infer causality. Third, the research only examines the effects of gender differences on pleasure without consideration of other factors. Fourth, self-report measurements were used in this study. Fifth, the indirect effects were significant in this study, and their effect sizes were small and only slightly lowered the direct path. However, it is the first study to explore the mechanism of association between gender differences and anticipatory and consummatory pleasure. This study was exploratory and has encouraged subsequent studies to continue to explore relevant mechanisms. Further studies need to use objective measures and control for relevant variables, in which the nature of the differences between females and males in the state of pleasure can be better understood.

## Conclusion

These results indicate that gender differences can positively predict pleasure, and cognitive flexibility and emotional expressivity play a partial mediating role between gender differences and anticipatory and consummatory pleasure. In the future, we should provide people with more platforms and opportunities, like mental health education and consultation, to assist them to express their emotions, especially negative emotions.

## Data Availability

The datasets generated and analyzed during the current study are not publicly available due to no permission from participants to share anonymized participant data publicly but are available from the corresponding author on reasonable request.

## Abbreviations

TEPs: The Temporal Experience of Pleasure Scale
CFI: The Cognitive Flexibility Inventory
BEQ: The Berkeley Expressivity Questionnaire
CI: Confidence interval
χ^2^: Chi-square
CFI: Comparative fit index
TLI: Tucker-Lewis index
RMSEA: Root mean square error of approximation
SRMR: Standardized root mean squared residual
